# Granzyme B in Autoimmune Skin Disease

**DOI:** 10.3390/biom13020388

**Published:** 2023-02-18

**Authors:** Anna Gleave, David J. Granville

**Affiliations:** 1 British Columbia Professional Firefighters’ Burn and Wound Healing Laboratory, International Collaboration On Repair Discoveries (ICORD) Centre, Vancouver Coastal Health Research Institute, University of British Columbia, Vancouver, BC V5Z 1M9, Canada; 2 Department of Pathology and Laboratory Medicine, University of British Columbia, Vancouver, BC V6T 1Z7, Canada

**Keywords:** Granzyme B, serine protease, autoimmune skin disease, inflammation, extracellular matrix, small molecule inhibitor

## Abstract

Autoimmune diseases often present with cutaneous symptoms that contribute to dysfunction, disfigurement, and in many cases, reduced quality-of-life. Unfortunately, treatment options for many autoimmune skin diseases are limited. Local and systemic corticosteroids remain the current standard-of-care but are associated with significant adverse effects. Hence, there is an unmet need for novel therapies that block molecular drivers of disease in a local and/or targeted manner. Granzyme B (GzmB) is a serine protease with known cytotoxic activity and emerging extracellular functions, including the cleavage of cell–cell junctions, basement membranes, cell receptors, and other structural proteins. While minimal to absent in healthy skin, GzmB is markedly elevated in alopecia areata, interface dermatitis, pemphigoid disease, psoriasis, systemic sclerosis, and vitiligo. This review will discuss the role of GzmB in immunity, blistering, apoptosis, and barrier dysfunction in the context of autoimmune skin disease. GzmB plays a causal role in the development of pemphigoid disease and carries diagnostic and prognostic significance in cutaneous lupus erythematosus, vitiligo, and alopecia areata. Taken together, these data support GzmB as a promising therapeutic target for autoimmune skin diseases impacted by impaired barrier function, inflammation, and/or blistering.

## 1. Background

Autoimmune diseases are a major cause of global morbidity and mortality [1,2,3]. Over the past three decades, the incidence and prevalence of autoimmune diseases have been increasing at an accelerated rate [3,4]. Because the onset of autoimmune diseases often occurs early in life and most are chronic in nature, the resulting disruption of working and reproductive capabilities incurs significant societal costs [5,6]. This has precipitated efforts to better characterize autoimmune disease pathophysiology, which despite recent advancements, remains poorly defined. Autoimmune diseases are thought to occur in individuals with a genetic predisposition to immune dysregulation following exposure to an environmental trigger [5]. Immune responses can be categorized into two distinct but frequently intertwined mechanisms [7]. The Th1 or “cytotoxic” response involves CD8+ T cells targeting intracellular antigens, causing apoptosis. Conversely, the Th2 or “humoral” response involves B cell proliferation and antibody generation in response to an extracellular antigen. In autoimmune diseases, these immune responses are erroneously directed against self-antigens, triggering a cascade of inflammatory cytokine release (IL-1β, IL-2, IL-12, TNF-α, and IFN-γ in the Th1 response; IL-4, IL-5, IL-10, and IL-13 in the Th2 response), immune cell recruitment, and inflammation with potentially permanent target tissue damage [7,8].

The skin is the largest organ in the human body and is impacted by many autoimmune diseases [9]. Several conditions, such as vitiligo, alopecia areata, and pemphigoid disease, primarily affect the integumentary system. Additionally, systemic autoimmune diseases often have characteristic skin findings. An estimated 80% of systemic lupus erythematosus (SLE) patients have cutaneous symptoms, ranging from the classic malar rash to permanent discoid scarring [10]. In systemic sclerosis, 50% of patients develop digital ulcers that predispose them to osteomyelitis, gangrene, and amputation [11]. Skin lesions can also precede other symptoms, creating opportunities for early diagnosis and intervention. For instance, 75% of patients with dermatitis herpetiformis have concurrent glucose-associated enteropathies, despite only 20% presenting with gastrointestinal symptoms [12]. Unfortunately, therapies for autoimmune dermatological diseases remain limited. Local and systemic corticosteroids are the current standard of care, but they can cause unpleasant and even life-threatening side effects [13,14,15]. Hence, there is an unmet need for novel therapies that block pathological disease drivers in a local and/or targeted manner.

Granzyme B (GzmB) is a serine protease produced by natural killer (NK) and CD8+ T cells and stored within cytotoxic intracellular granules [16,17,18]. GzmB has well-defined intracellular activity; following granule secretion, GzmB is internalized into target cells with the help of the pore-forming protein, perforin, where it initiates apoptosis through caspase-dependent and -independent mechanisms [18,19,20]. Interestingly, during this process, GzmB may also leak into the extracellular space [21]. More recently, extracellular functions for Granzyme B have been recognized that expand its role beyond cytotoxicity [17,22,23]. For instance, GzmB is produced by multiple immune and non-immune cell types that do not express perforin, including mast cells [24], basophils [25], neutrophils [26], B cells [27], plasmacytoid dendritic cells [28], macrophages [29], CD4+ T cells [30], regulatory T cells [31], and keratinocytes [32]. GzmB released from the aforementioned cell types accumulates in the extracellular space, where it retains proteolytic activity, and depending on location and available substrates, cleaves extracellular matrix proteins, attachment/barrier proteins, complement factors, coagulation factors, and augments cytokine/growth factor activity, among other actions [21,33,34].

In humans, GzmB is encoded by the *GZMB* gene, which is 3500 base pairs long, comprises 5 exons and 4 introns, and is located on chromosome 14 [35]. Transcriptional activation of the *GZMB* gene is mediated by a combination of T cell activation and cytokine co-stimulation [36]. Once transcribed, the 32 KDa, 247 amino acid polypeptide is targeted to the endoplasmic reticulum and cleaved into an inactive pro-enzyme [23]. GzmB is then tagged with mannose-6-phosphate in the Golgi, which promotes internalization [23]. The tertiary structure of GzmB involves eight loops grouped into two domains with the active site situated in between [37]. The active site contains an Arg residue, which interacts strictly with substrates containing Asp, or less preferentially Glu, in the P1 position [37,38]. Finally, GzmB is activated through the cleavage of the N-terminal dipeptide by cathepsin C [23]. While proteolytic activity is suppressed within the acidic granules, subsequent release into a pH-neutral environment enables optimal function [38]. The only known endogenous intracellular GzmB inhibitor is proteinase inhibitor-9 (SerpinB9), which is produced by immune cells and serves as protection in the event of accidental GzmB release [39]. To date, however, no extracellular endogenous GzmB inhibitors have been identified. Several exogenous GzmB inhibitors have also been developed, including Compound 20 [40,41], Z-IETD-fluoromethylketone [42,43], and VTI-1002 [33,44,45] among others. Only VTI-1002 has been studied in preclinical models of autoimmune skin disease, showing no adverse effects for up to 30 days of daily administration, but has not yet been examined in clinical trials [34].

**Figure 1:**
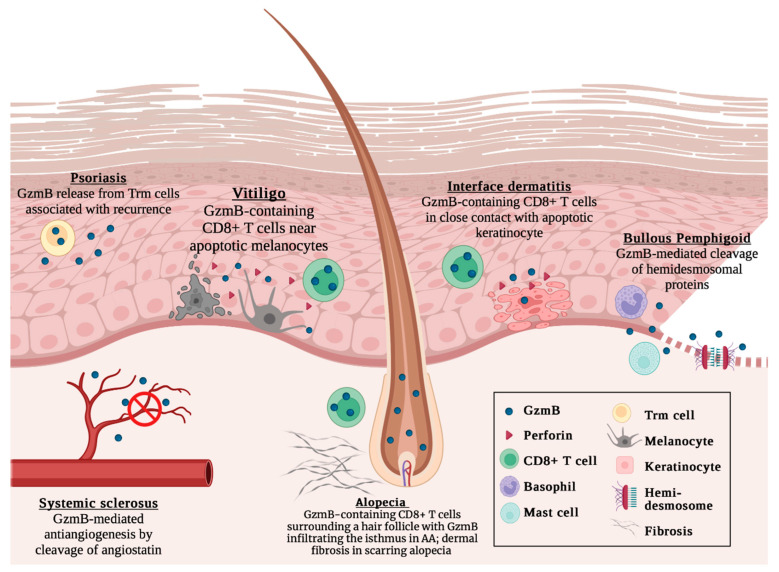
Current understanding of GzmB in cutaneous autoimmune diseases, including psoriasis, vitiligo, systemic sclerosis, alopecia areata, interface dermatitis, and bullous pemphigoid. Not to scale. Created with BioRender.com, accessed on 4 January 2023.

While virtually absent in healthy skin, several autoimmune skin diseases exhibit elevated GzmB levels. This suggests a lack of physiologic function for GzmB, rendering it an attractive therapeutic target for minimizing adverse effects [46,47,48,49]. In this review, we summarize our current understanding of GzmB and its contributions to autoimmune skin disease pathophysiology, including skin inflammation, cell apoptosis, and structural protein disruption. We discuss a broad range of autoimmune skin diseases, such as alopecia areata, interface dermatitis, pemphigoid disease, psoriasis, systemic sclerosis, and vitiligo, among others (Figure 1). A causal relationship has been established between GzmB levels and the onset of pemphigoid disease, with GzmB inhibition with VTI-1002 effectively reverting symptoms [34,40]. GzmB may also carry diagnostic and prognostic significance in cutaneous lupus erythematosus [50,51,52], vitiligo [53,54], and alopecia areata/scarring alopecia [55,56,57]. Collectively, the current evidence nominates GzmB as a promising biomarker and therapeutic target in autoimmune skin disease.

## 2. Alopecia Areata

Alopecia areata (AA) is characterized by transient, non-scarring hair loss secondary to immune privilege collapse within the hair follicle [58]. The extent of hair loss ranges from well-circumscribed patches to complete loss from all hair-bearing sites [58]. AA immunopathogenesis is not currently well understood but is thought to involve hair follicle autoantigen recognition, which triggers CD8+ T cell recruitment, J

**Figure 2:**
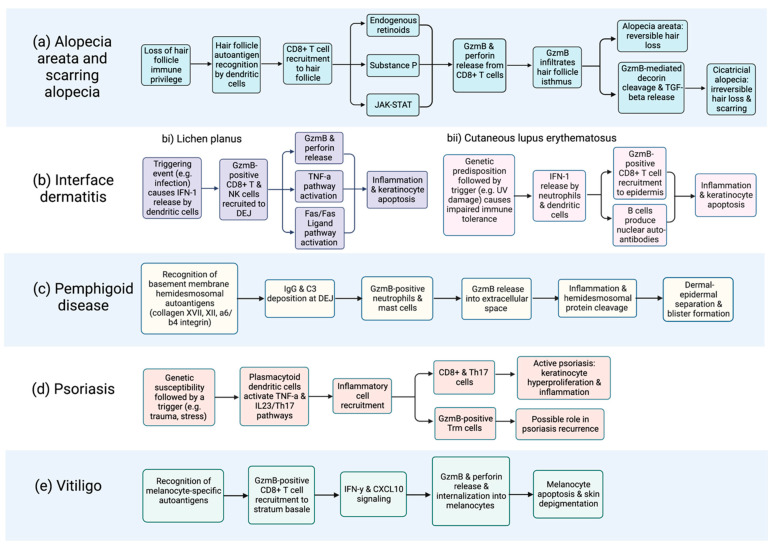
Flow chart depicting proposed GzmB involvement in the immunopathogenesis of cutaneous autoimmune diseases, including (**a**) alopecia and scarring alopecia; (**b**) interface dermatitides such as (**bi**) lichen planus and (**bii**) cutaneous lupus erythematosus; (**c**) pemphigoid disease; (**d**) psoriasis; and (**e**) vitiligo. Of note, the contribution of extracellular GzmB to disease onset and severity requires further elucidation and is likely underrepresented in this figure. Characterization of the extracellular functions of GzmB is an area of ongoing investigation. Systemic sclerosis is not included due to the absence of relevant data. Created with BioRender.com, accessed on 17 February 2023.

AK-STAT pathway activation, and perforin and GzmB release (Figure 2a) [59,60,61,62]. Other pathways, including endogenous retinoids [63,64,65] and substance P [65,66], are functionally linked to AA and may increase GzmB levels. GzmB has been proposed as a downstream mediator in the JAK/STAT pathway in AA, as the Jak3 inhibitor tofacitinib was observed to downregulate GzmB in AA-affected skin cells in a mouse model [59]. Moreover, GzmB-expressing CD8+ T cells exist in close contact with affected hair follicles [67]. GzmB has even been detected within the hair follicle isthmus, the primary site of immune collapse [63]. A temporal association between hair loss and GzmB expression has also been established using in vivo models. McPhee et al. [68] transplanted skin grafts from the C3H/HeJ mouse model into normal mice, which stimulated hair loss and increased GzmB expression. Similarly, Hashimoto et al. [69] injected cryopreserved lymphocytes from C3H/HeJ mice into control mice, which caused 90% of recipient mice to develop hair loss and marked upregulation of GzmB mRNA. While these data implicate GzmB in AA pathogenesis, a causal relationship has not been established.

GzmB expression in AA may also predict disease severity and treatment response. Koguchi-Yoshioka et al. [55] compared lymphocyte phenotypes in AA lesions from responders and non-responders to squaric acid dibutyl ester, a topical immunotherapy for refractory AA. Skin lesions from responders, non-responders, and controls all had similar numbers of GzmB-expressing lymphocytes. However, individual lymphocytes from non-responders produced significantly more GzmB than responders and controls. This was observed in skin lesions but not in the serum, suggesting that topical GzmB inhibition may effectively treat AA while avoiding systemic side effects. AA is one of the most prevalent autoimmune conditions [70]; despite this, little consensus exists regarding treatment due to arbitrary reference cut-offs and variable results from randomized trials [71,72]. The finding that GzmB acts locally as a downstream mediator of inflammation highlights its potential as a therapeutic target in AA.

Unlike AA, in which hair follicle integrity is preserved, scarring or cicatricial alopecia involves permanent follicular destruction and hair loss [73]. The resultant scarring is irreversible and causes profound injury to patient quality-of-life [74]. As such, efforts to elucidate the mechanisms that drive scarring are ongoing. In one study, Ghoreishi et al. [56] noted an increased number of GzmB-positive CD8+ and NK cells in cicatricial alopecia compared to AA, indicating a greater extent of T cell activation. Similar findings were observed in frontal fibrosing alopecia, a subtype of scarring alopecia with progressive frontotemporal and eyebrow hair loss [57]. GzmB is also known to cleave decorin, an anti-fibrotic proteoglycan that sequesters active transforming growth factor beta (TGF-β) [75]. Keloid and hypertrophic scars contain reduced decorin, suggesting that GzmB may mediate scarring through decorin cleavage and subsequent TGF-β release [76,77,78]. GzmB inhibitors serpina3n and VTI-1002 prevent degradation of extracellular decorin in vivo and, therefore, represent attractive anti-fibrotic therapies [44,79]. However, the role of the decorin-TGF axis in cicatricial alopecia remains uncertain. Some have observed elevated TGF-β in cicatricial alopecia [80] while others report unchanged [81] and even reduced [82] TGF-β levels. The impact of GzmB on the decorin-TGF axis in scarring alopecia should be further explored.

## 3. Interface Dermatitis

Interface dermatitis comprises a diverse group of skin disorders characterized by keratinocyte apoptosis and pathologic inflammation within the dermal-epidermal junction (DEJ) [83]. A classic feature of interface dermatitis is the formation of colloid bodies, which reflect intraepidermal apoptosis [84]. Lichen planus is the prototypical example of interface dermatitis, although the term also includes systemic illnesses, such as systemic lupus erythematosus (SLE) and dermatomyositis. These conditions are thought to possess a shared inflammatory signaling pathway involving the recognition of self-antigens on keratinocytes, followed by CD8+ and NK cell recruitment to the DEJ, and finally keratinocyte apoptosis within the stratum basale [85]. The triggering self-antigens vary by condition; for example, Ro/SSA antigen may act as a specific target in SLE while allogenic MHC may play a role in graft-versus-host disease [86]. GzmB release from CD8+ and NK cells is a common inducer of keratinocyte cell death across the interface dermatitides, although TNF-alpha and Fas/Fas Ligand systems may also play a role [48].

Lichen planus (LP) is a chronic autoimmune condition with cutaneous and oral manifestations that classically presents with violaceous plaques and a white lacelike pattern termed Wickham’s striae [87]. The triggering event is thought to involve IFN-1 release from plasmacytoid dendritic cells, which promotes inflammatory cell recruitment to the DEJ (Figure 2bi) [88]. The inflammatory infiltrate contains CD8+ T cells that stain strongly for GzmB and perforin, which is not observed in healthy skin [47,48,88,89]. Ammar et al. [90] demonstrated that GzmB levels are 200 times higher in LP than in healthy skin, with higher expression observed in oral compared to cutaneous LP [48]. The number of GzmB-expressing cells is highest early in the disease and eventually plateaus, suggesting that GzmB plays a role in early LP pathogenesis. Notably, a correlation has been established between the number of lymphocytes expressing GzmB and the extent of keratinocyte death [91]. This is supported by Lage et al. [48], who observed GzmB-containing T lymphocytes in close proximity to apoptotic keratinocytes. Similarly, Shimizu et al. [89] reported GzmB release from T cells into the intracellular space directly adjacent to apoptotic keratinocytes. While association has been established, the impact of GzmB inhibition on disease onset and severity has yet to be studied in LP.

Systemic lupus erythematosus (SLE) is a chronic autoimmune disorder that can affect nearly every organ system. Approximately 80% of SLE patients have cutaneous symptoms known as cutaneous lupus erythematosus (CLE) [10]. Symptoms can be highly heterogenous, ranging from systemic involvement to the classic malar rash and photosensitivity in acute presentations, and chronic disfiguring scarring in discoid lupus erythematosus (DLE) [92]. Immunopathogenesis is thought to occur in genetically susceptible individuals following an environmental trigger, such as ultraviolet damage. This triggers an IFN signaling loop, cytokine release, and immune cell infiltrate, including CD8+ T cells and autoantibody-producing plasma cells (Figure 2bii) [93,94]. Current treatment options include hydroxychloroquine and methotrexate, which are effective but have well-documented adverse effects [14]. GzmB has been implicated in the pathogenesis of CLE, with one study detecting GzmB in histological skin specimens in 77% of affected patients [84]. Moreover, CD8+ lymphocytes in CLE produce GzmB-specific autoantigens, a finding that is abolished by the addition of a GzmB inhibitor [50,95]. Biopsies of DLE stain strongly for GzmB relative to nonscarring CLE and controls, with expression levels correlating to clinicopathological severity [96,97]. Others have arrived at similar conclusions, linking GzmB expression to both disease severity, duration and/or chronicity [50,51]. Another study on a large cohort of SLE patients found higher levels of serum GzmB in patients with cutaneous symptoms [52]. Some have even linked the presence of GzmB in skin biopsies and serum with poor renal function, including elevated creatinine and proteinuria [51,96]. These studies suggest that GzmB is a key driver in the pathogenesis of SLE and may correlate with disease severity.

Dermatomyositis (DM) is a chronic autoimmune myopathy with distinct cutaneous manifestations, including heliotrope rash and Gottron’s papules [98]. Conflicting evidence exists regarding the role of GzmB in DM. For instance, Grassi et al. [84] compared GzmB staining in histological skin specimens of DM and SLE. They observed strong staining in only 2 of 20 DM specimens, whereas 17 of 22 SLE specimens stained strongly for GzmB. The Fas/Fas Ligand pathway has been alternatively proposed to drive keratinocyte apoptosis in DM based on the finding of strong Fas staining within the epidermis of Gottron’s papules [99]. Conversely, other studies have implicated GzmB in DM. Goebels et al. [100] noted increased GzmB staining in DM while Mammen [101] identified a protein called Jo-1, which correlates with DM disease severity and is cleaved by GzmB in the lungs. Not unlike other autoimmune conditions, the roles of GzmB in DM, and particularly its extracellular roles, remain unclear and should be further elucidated.

## 4. Pemphigoid Diseases

Pemphigoid diseases encompass bullous pemphigoid, epidermolysis bullosa acquisita, and dermatitis herpetiformis. These conditions are characterized by tense, pruritic subepidermal bullae secondary to autoantibody targeting of extracellular structural proteins within the DEJ basement membrane (Figure 2c) [102]. Subsequent separation between the dermis and epidermis causes blistering, erythema, and erosion. Bullous pemphigoid (BP), the most common autoimmune blistering disease, is becoming more prevalent owing to an aging population and more frequent use of triggering medications [103]. Potent steroids are effective in treating BP, but their use, especially long-term, is limited by significant side effects [104]. Adverse effects include skin atrophy and impaired wound healing locally and systemic side effects, such as decreased bone density and Cushing’s syndrome, which can be especially problematic in the medically vulnerable elderly population [105]. Therefore, the development of novel targeted therapies for pemphigoid diseases is an unmet clinical need. Prior research on GzmB in pemphigoid disease is scant owing to the former belief that GzmB functioned as an exclusively intracellular protease. In light of new research revealing an extracellular function for GzmB, one involving extracellular matrix and hemidesmosomal protein cleavage, the role of GzmB in pemphigoid disease has received increased attention [34,40,106].

GzmB has been shown to be elevated in the immune cells and extracellular milieu in pemphigoid diseases. Furthermore, GzmB proteolysis likely plays a key role in cleaving structural proteins that are critical for maintaining DEJ integrity. For example, hemidesmosomal proteins Collagen XVII, Collagen VII, and α6/β4 integrin are known autoantibody targets in pemphigoid diseases and substrates of GzmB [34,40,107]. Based on these findings, a causal relationship between GzmB and pemphigoid disease has been investigated. Russo et al. [40] performed an immunohistochemical analysis of BP, epidermolysis bullosa acquisita, and dermatitis herpetiformis specimens, and they observed GzmB-specific cleavage of Collagen XVII, Collagen VII, and α6/β4 integrin. Blister formation in diseased specimens was indicated histologically by the presence of clefts separating the dermis and epidermis. This was accompanied by GzmB accumulation at the DEJ, along with a paucity of Collagen XVII, Collagen VII, and α6/β4 integrin. Importantly, the incubation of GzmB with healthy skin samples produced a similar cleft-forming phenotype, which was abolished following GzmB inhibition with Compound 20. To explore a causal, mechanistic role in vivo, Hiroyasu et al. [34] utilized a murine model of BP, as well as local and systemic antibody-transfer murine models of epidermolysis bullosa acquisita, to assess the role of GzmB via either GzmB knockout or topical pharmacological inhibition with VTI-1002. GzmB was produced by mast cells and basophils, which do not produce perforin, further suggesting an extracellular function [34]. GzmB inhibition led to a 45% reduction in lesional area; prevented cleavage of Collagen XVII, Collagen VII, and α6/β4 integrin; and markedly reduced skin frailty compared to the controls. Moreover, an analysis of GzmB levels in human BP patients via ELISA revealed that GzmB was significantly elevated in BP blister fluids but negligible in BP and control sera [34]. These findings imply a causal role for extracellular (not intracellular/perforin-dependent) GzmB in pemphigoid disease pathogenesis. Further exploration of therapeutic approaches that target extracellular GzmB for pemphigoid diseases is of significant interest.

## 5. Psoriasis

Psoriasis is a chronic, complex, and relapsing skin condition affecting 3% of the population [108]. The most prevalent subtype, psoriasis vulgaris, is characterized by immune cell infiltration and hyperproliferation of abnormal keratinocytes within the epidermis [109]. Clinical features include the formation of erythematous, pruritic, and scaly plaques affecting the trunk, scalp, and extensor body surfaces [109]. The TNF-α and IL-23/TH17 pathways are critical drivers of psoriasis development and chronicity (Figure 2d) [110]. These pathways are targeted by several biologic therapies that provide highly effective disease management [110]. At present, the role of GzmB in psoriasis pathophysiology is poorly understood. While GzmB is elevated in psoriasis compared to healthy controls, no correlation has been established between GzmB expression and clinical severity [111,112]. An alternate role for GzmB has been proposed by Fenix et al. [113], who used mouse models to study CD49a+ resident memory T (Trm) cells associated with site-specific disease memory in psoriasis. Comparison across acute, chronic, and resolved lesions showed significantly increased GzmB production by Trm cells in resolved psoriasis. This led the authors to speculate that GzmB contributes to relapsing rather than acute disease. Because biologics can be prohibitively expensive, drug tapers are often attempted despite poorly defined tapering regimens, resulting in disease re-occurrence [114,115]. The exploration of GzmB inhibition as a means for maintaining psoriatic remission may therefore be warranted.

## 6. Systemic Sclerosis

Systemic sclerosis, also known as scleroderma, is a fibrogenic rheumatic condition that causes progressive sclerosis of the skin and internal organs, as well as vasculopathy [116]. Albeit uncommon, systemic sclerosis bears the highest mortality rate of any rheumatic disease [116]. Given its infrequency, high mortality, and breadth of organ systems affected, establishing determinants of distinct clinical subsets would be beneficial for guiding risk stratification and therapy. Evidence suggests that GzmB is elevated in systemic sclerosis and may be associated with certain disease complications [117]. For example, Ulanet et al. [118] demonstrated that B23, a nucleolar phosphoprotein associated with pulmonary hypertension in scleroderma, was cleaved efficiently by GzmB in vascular smooth muscle cells. Interestingly, other cell lines were highly resistant to B23 cleavage by GzmB. The authors, therefore, proposed that autoantigens generated by cleavage from tissue-specific proteases, including GzmB, induce different disease subtypes and represent targets for individualized therapies.

GzmB has also been investigated in other scleroderma-associated vasculopathies. It is well known that plasminogen cleavage paradoxically produces both proangiogenic plasmin and antiangiogenic angiostatin. Mulligan-Kehoe et al. [119] analyzed the serum of scleroderma patients and noted elevated angiostatin along with reduced plasmin concentrations. Furthermore, GzmB incubation with plasminogen and plasmin produced angiostatin fragments. This led the authors to speculate that GzmB may account for some of the vascular defects observed in scleroderma via the generation of antiangiogenic factors like angiostatin. Another study considered ischemic digital loss, a complication of vasculopathy seen in systemic sclerosis. Schachna et al. [120] performed immunoblots with cell lysates from scleroderma patients with and without digital loss; staining for GzmB autoantigens was positive in 84% of patients with digital loss, compared to 40% without digital loss. Hence, the presence of specific autoantigens cleaved by GzmB carries clinical relevance, as it may predict the development of complications like ischemic digital loss. In fact, it has been proposed that GzmB-mediated autoantigen cleavage occurs in the majority of autoimmune diseases, with generated peptide fragments contributing to a feed-forward loop that drives disease self-sustenance [121]. Further investigation into autoantigen fragments that may serve as biomarkers for other clinical subtypes would be a topic of future interest.

## 7. Vitiligo

Vitiligo is a chronic depigmenting disorder resulting from an autoimmune attack against melanocytes, the cell type responsible for producing melanin. It affects 0.2% of the population and is characterized by the presence of well-defined nonpigmented macules and patches [122,123]. Autoimmunity is driven by the recognition of melanocyte-specific autoantigens, CD8+ T cell recruitment, and activation of the IFN-γ-CXCL10 cytokine signaling pathway (Figure 2e) [124]. While phototherapy and corticosteroids are effective short-term therapies for vitiligo, they are time-consuming, require long-term follow-up, and may not provide longstanding symptomatic control [13]. To address these challenges, the identification of biomarkers for disease prognostication and therapeutic targeting would be invaluable. Several single nucleotide polymorphisms in *GZMB*, the gene encoding GzmB, have been associated with vitiligo [125,126,127]. In fact, one study, including over 3000 patients of Chinese Han ancestry, associated the SNP rs8192917 C allele with a 40% increased risk of developing vitiligo [128]. These findings suggest that GzmB is an important genetic locus in vitiligo, although this should be further investigated in studies incorporating subjects from more diverse ethnic backgrounds.

In addition to conferring a genetic predisposition to vitiligo, GzmB may offer clinical relevance as a diagnostic and prognostic tool. Immunohistochemical analysis has shown localization of GzmB-containing CD8+ T cells along the edges of vitiligo lesions in direct contact with apoptotic melanocytes [46]. These findings were absent in lesional, non-lesional, and healthy control specimens. Others have similarly observed elevated expression of GzmB in vitiligo patients’ skin biopsies, plasma samples, or both [54,129,130,131]. Moreover, a complete cytokine panel by Ng et al. [54] ranked GzmB among the top three predictive biomarkers for disease severity. This was consistent with Bertolotti et al. [53], who noted more profound infiltration of GzmB-expressing T cells in very progressive vitiligo. In spite of evidence nominating GzmB as a potential predictive biomarker and therapeutic target in vitiligo, causality has not been established. The effects of GzmB knockout or inhibition in vitiligo should be further examined.

## 8. Conclusions

Despite having a high prevalence and morbidity, cutaneous autoimmune diseases lack accessible and effective therapies with reasonable side effect profiles. While previously recognized exclusively for its pro-apoptotic function, GzmB has emerged as a key pathologic initiator of extracellular processes ranging from dysregulated growth factor release and inflammation to desmosomal/hemidesmosomal cleavage and/or fibrosis. GzmB elevations accompany many autoimmune skin diseases and may play a driving role in the onset and progression of the disease. These findings reflect the central role of GzmB as a downstream mediator of several pathways involved in skin autoimmunity. Since GzmB is not significantly observed in the extracellular milieu under normal conditions, it represents an attractive therapeutic target for minimizing off-target effects. In spite of this, there remains a need to assess viable drug candidates in the clinic. Future efforts should focus on advancing clinical trials examining the safety and efficacy of GzmB inhibition and the generation of GzmB-based diagnostic and prognostic tools. It is important to note that, while extracellular GzmB contributes to impaired barrier function, the cleavage of basement membrane/dermal-epidermal junction proteins, vascular permeability, and inflammation, the role(s) of extracellular GzmB in autoimmune pathologies is still in its infancy and not yet fully characterized. Consequently, while GzmB is abundant in many autoimmune skin pathologies, the majority of previous studies have not considered the recently discovered extracellular roles for this protease. Given that GzmB retains its activity in the extracellular milieu, it is likely that such mechanisms could contribute to the onset and progression of disease.

## Data Availability

Not applicable.
